# Igniting the slow burn of post-COVID conditions

**DOI:** 10.1128/mbio.01489-23

**Published:** 2023-09-26

**Authors:** Jeffrey P. Henderson

**Affiliations:** 1 Department of Medicine, Division of Infectious Diseases, Washington University School of Medicine, St. Louis, Missouri, USA; 2 Center for Women’s Infectious Disease Research, Washington University School of Medicine, St. Louis, Missouri, USA; Griffith University-Gold Coast Campus, Gold Coast, Queensland, Australia

**Keywords:** COVID-19, post-COVID conditions, convalescent plasma, antiviral agents

## Abstract

Post-COVID conditions (PCCs) are persistent new patient symptoms occurring after acute COVID-19 infection and are an increasingly appreciated dimension of the COVID-19 pandemic. The factors that cause PCCs are not well understood. In recent work, Gebo et al. identify a connection between acute IL-6 levels, early COVID-19 convalescent plasma (CP) administration, and later PCCs in subjects from a randomized controlled trial of acutely ill subjects enrolled in 2020 to 2021 (K. A. Gebo, S. L. Heath, Y. Fukuta, X. Zhu, et al., mBio e00618-23, 2023, https://doi.org/10.1128/mbio.00618-23). These results may be viewed as part of an emerging picture linking the intensity of inflammatory responses during acute infection to later PCCs.

## COMMENTARY

In the 3 years since the COVID-19 pandemic arrived, medical consideration of this illness has broadened from an initial focus on surviving acute SARS-CoV-2 infection to now including attempts to better understand and impact the long-term sequelae of COVID-19. Post-COVID conditions (PCCs) most typically manifest as fatigue, shortness of breath, and cognitive dysfunction that persist for weeks after resolution of the acute infection (1, 2). Among the possible instigators of PCCs are autoimmunity generated during initial encounter with the virus or the sequelae of more general inflammatory tissue damage. A useful organizing principle for considering acute COVID-19 is the damage-response framework, which recognizes the temporal progression from initial encounter with SARS-CoV-2 to the subsequent inflammation responsible for most deaths (3). In patient studies, one way to differentiate the origins of PCCs is to assess the effects of therapies administered after SARS-CoV-2 infection that alter this progression.

In recent work in *mBio*, Gebo et al. analyze data from a randomized controlled trial conducted prior to widespread vaccination to determine if early COVID-19 convalescent plasma (CP) administration affects the development of PCCs (4). CP contains antibodies against SARS-CoV-2 from donors that have recovered from COVID-19 and diminish progression to severe disease when given sufficiently early (5 – 7). The authors found that subjects receiving early CP treatment (<5 days from symptom onset) had lower odds of PCC 90 days after the start of symptoms than subjects receiving CP later. PCC in the patient cohort was also associated with elevated IL-6 during acute infection as well as female sex and older age.

The determinative role of early CP infusion timing corresponds to multiple studies showing that effective antiviral therapy for non-immunocompromised patients with COVID-19 must be initiated early in illness (5 – 7). Early antiviral therapy with CP dampens the subsequent immune response to infection, lowering the risk of respiratory failure and death (8). The association between PCCs and baseline IL-6 further implicates pro-inflammatory responses as a likely mechanism of CP benefit in this cohort. Similar findings have recently been reported in observational studies of small molecule antiviral agents, consistent with the antiviral mechanism of action for CP (9, 10). Interestingly, a similar protective effect has been observed in an early treatment trial with metformin, a repurposed drug for metabolic disease recently associated with SARS-CoV-2 antiviral activity (11).

Relationships between PCCs and immunomodulatory agents (corticosteroids and monoclonal antibodies directed against pro-inflammatory mediators), a second class of COVID-19 therapeutics (12), remain unclear. In contrast with antiviral therapies, these agents may confer benefit only in patients with severe COVID-19, where they appear to exert their protective effect by dampening damage from an exuberant inflammatory response. Use of these agents outside late or severe disease could be harmful, either by predisposing to opportunistic infections or by permitting prolonged SARS-CoV-2 viremia. Indeed, corticosteroid use may diminish the benefit from CP (13). One small prospective cohort study found no effect of corticosteroid treatment on PCCs (14). The absence of further reports may reflect difficulty in assembling an adequate study or the persistent publication bias against null results (15). It is possible that, in the best candidates for immunomodulatory therapy, the die has been cast and PCC risk will be elevated regardless of therapy.

At present, individuals with a vaccine or convalescent immunity may be protected from PCCs compared to 2020 (16, 17), though this protection could wane for a future coronavirus that is more immunologically distinct. Patients with humoral immune deficiencies, who appear to benefit from CP for an extended period after initial infection (18, 19), may derive particular protection from PCCs with CP therapy. The study of PCCs in these patients may yield additional new insights.

While much more remains to be learned about PCCs, data from this therapeutic trial and from retrospective studies paint a picture consistent with the intensity of inflammatory responses during acute SARS-CoV-2 infection as an important contributor. As such, it is possible that patients with PCC share pathophysiologic features with survivors of sepsis who have long-term sequelae (20). This study illustrates the value of launching well-designed studies with prolonged follow-up early in the course of a new pandemic illness, when the course of disease may not be immediately apparent. Not only do these yield data in a timely manner, they also provide results in an immunologically naïve population where therapeutic effects are more profound.
